# Characterization of DNA Repair Foci in Root Cells of *Arabidopsis* in Response to DNA Damage

**DOI:** 10.3389/fpls.2019.00990

**Published:** 2019-07-30

**Authors:** Takeshi Hirakawa, Sachihiro Matsunaga

**Affiliations:** Department of Applied Biological Science, Faculty of Science and Technology, Tokyo University of Science, Chiba, Japan

**Keywords:** DNA damage response, DNA repair, homologous recombination, RAD54, nuclear envelope

## Abstract

As a sessile organism, plants are constantly challenged by diverse environmental stresses that threaten genome integrity by way of induction of DNA damage. In plants, each tissue is composed of differentiated cell types, and the response to DNA damage differs among each cell type. However, limited information is available on the subnuclear dynamics of different cell types in response to DNA damage in plants. A chromatin remodeling factor RAD54, which plays an important role in the exchange reaction and alteration of chromatin structure during homologous recombination, specifically accumulates at damaged sites, forming DNA repair foci (termed RAD54 foci) in nuclei after γ-irradiation. In this study, we performed a time-course analysis of the appearance of RAD54 foci in root cells of *Arabidopsis* after γ-irradiation to characterize the subnuclear dynamics in each cell type. A short time after γ-irradiation, no significant difference in detection frequency of RAD54 foci was observed among epidermal, cortical, and endodermal cells in the meristematic zone of roots. Interestingly, cells showing RAD54 foci persisted in roots at long time after γ-irradiation, and RAD54 foci in these cells localized to nuclear periphery with high frequency. These observations suggest that the nuclear envelope plays a role in the maintenance of genome stability in response to DNA damage in *Arabidopsis* roots.

## Introduction

Genome integrity is constantly threatened by exogenous (e.g., ionizing radiation, ultraviolet light, and chemical components) and endogenous stresses (e.g., stalled DNA replication forks and reactive oxygen species) that induce DNA damage in organisms. In plants, DNA damage is also caused by diverse environmental stresses, such as stress-mediated reactive oxygen species, pathogen infection, high boron concentration, and aluminum ions (Rounds and Larsen, 2008; Sakamoto et al., 2011; Baxter et al., 2014; Song and Bent, 2014). Signaling of DNA damage is rapidly coordinated with several mediators to maintain genome stability in plants. In response to DNA damage in plants, ataxia telangiectasia mutated (ATM) and ATM/rad3-related kinases, which are sensor proteins for DNA double-strand breaks (DSBs) and single-strand DNA, respectively, activate the SUPPRESSOR OF GAMMA RESPONSE 1 (SOG1) transcription factor through phosphorylation (Culligan et al., 2004; Yoshiyama et al., 2013a). The active form of SOG1 directly regulates expression of genes participating in DNA repair, cell cycle progression, pathogen response, and phytohormone signaling (Ogita et al., 2018).

After the induction of DSBs, programmed cell death (PCD) is induced specifically in stem cells of the root meristematic zone and the central zone of shoot apical meristems in *Arabidopsis* (Fulcher and Sablowski, 2009). In contrast, the quiescent center (QC) cells, which maintain the homeostasis of stem cells, do not show PCD or morphological alterations in roots with DSBs. In the epidermis and cortex of roots, DSBs induce both arrest of the cell cycle and endoreduplication. Endoreduplication is triggered by inhibiting G2/M progression and specialized cell cycle where DNA replication is repeated without mitosis and cytokinesis, following expansion of the cell volume (Adachi et al., 2011). These findings suggest that the cellular response to DNA damage differs among each cell type in roots following DNA damage. However, little is known about the subnuclear dynamics in each cell type during the response to DNA damage.

In response to DNA damage, DNA repair foci, which are the subnuclear foci formed by DNA repair factors that accumulate specifically at damaged sites, are detected as distinct spots in nuclei (Rothkamm et al., 2015). A phosphorylated histone variant H2AX, termed γH2AX, which is detected around damaged sites and functions as a marker recruiting other DNA repair factors, forms several subnuclear foci upon DNA damage (Rogakou et al., 1999). In plants, the phosphorylation of H2AX is downstream of the activation of ATM by DSBs, and the detection frequency of γH2AX foci increases in a dose-dependent manner following induction of DSBs (Friesner et al., 2005). Thus, γH2AX foci are used as tools to measure DNA repair activity in plant cells upon DNA damage. However, γH2AX foci are undetectable in living cells because immunostaining using a specific antibody is involved. Several studies have shown that certain DNA repair factors form DNA repair foci in living cells of *Arabidopsis* in response to DNA damage (Lang et al., 2012; Jia et al., 2016; Biedermann et al., 2017; Horvath et al., 2017; Liu et al., 2017). Previously, we observed that the chromatin-remodeling factor RAD54, which regulates the spatiotemporal arrangement of homologous loci with DSBs, accumulates specifically at damaged sites, resulting in formation of DNA repair foci termed RAD54 foci (Hirakawa et al., 2015, 2017). RAD54 plays an important role in strand exchange and the alteration of chromatin structure during homologous recombination (HR) repair in eukaryotes (Heyer et al., 2010). *In vitro* analysis showed that yeast RAD54 has an activity of unwinding duplex DNA to promote the exchange reaction in HR (Mazin et al., 2000). In addition, human RAD54 slides nucleosomes along chromatin in an ATP-dependent manner to promote the homology search during HR *in vitro* (Zhang et al., 2007). The *Arabidopsis rad54* mutant shows low HR repair activity and high sensitivity to several genotoxic stresses (Osakabe et al., 2006), and as a result, RAD54 foci contribute to the progression of HR repair. In the present study, we monitored the formation of RAD54 foci in each cell type in *Arabidopsis* roots after the induction of DSBs to characterize the subnuclear dynamics following DNA damage of these cells.

## Materials and Methods

### Plant Materials and Growth Conditions

All plants used in this study were *Arabidopsis thaliana* ecotype Col-0. Transgenic plants expressing RAD54-EYFP with the *rad54-1* background were constructed in our previous study (Hirakawa et al., 2017). The double-mutant *crwn1/4* was used in a previous study (Sakamoto and Takagi, 2013). Sterilized seeds were sown on half-strength Murashige and Skoog (1/2 MS) medium plates (supplemented with 1% sucrose and 1% agar). After incubation at 4°C for 24 h, the plates were placed in an incubator maintained at 22°C with a 16/8 h (light/dark) photoperiod.

### γ-Irradiation and Microscopy

Five-day-old seedlings were exposed to 100 Gy γ-irradiation using a ^137^Cs source at a dose rate of 0.762 Gy/min at the Research Institute for Biomedical Science, Tokyo University of Science. After γ-irradiation, the roots were observed with a FV1200 confocal microscope equipped with a GaAsP detector (Olympus). To stain the cell walls, seedlings were immersed in 10 μg/ml propidium iodide/D.W. (Sigma-Aldrich) for 2 min. The detection frequency was obtained by dividing the cells showing RAD54 foci by RAD54 positive cells.

### EdU and DAPI Staining

Detection of 5-ethynyl-2′-deoxyuridine (EdU) was performed with the Click-iT^®^ Plus EdU Alexa Fluor^®^ 594 Imaging Kit (Thermo Fisher Scientific) in accordance with the manufacturer’s instructions. Five-day-old seedlings were exposed to 100 Gy γ-irradiation. After 24 h, the seedlings were incubated in liquid 1/2 MS medium containing 10 μM EdU for 20 min to specifically label cells during S phase at that time. The seedlings were fixed with 4% (w/v) paraformaldehyde/PBS for 40 min, washed in PBS, and then incubated in 0.5% (w/v) Triton X-100/PBS for 20 min. The samples were washed in PBS twice and incubated in the Click-iT reaction cocktail for 30 min in the dark. The Click-iT reaction cocktail was removed, and the samples were washed in PBS three times. The samples were washed in PEMT (50 mM PIPES, 2 mM EGTA, 2 mM MgSO_4_, 0.5% Triron X-100) buffer three times for 5 min each, and then washed in PBS. The samples were incubated in a mixture of DNA-staining solution (Sysmex)/PBS (3:1, v/v) for 3 min and then washed in PBS three times for 5 min each. The samples were mounted under a cover glass with 25% (v/v) 2,2′-thiodiethanol/PBS. Samples were observed with a FV1200 confocal microscope equipped with a GaAsP detector.

### Immunostaining

Immunostaining was performed as previously described (Hirakawa et al., 2017). Root tips of 5-day-old seedlings sampled 8 h after γ-irradiation (100 Gy) were analyzed. Rabbit anti-γH2AX (Hirakawa et al., 2017) was used as the primary antibody and diluted 1:100. Anti-rabbit Alexa Fluor 488 (Thermo Fisher Scientific) was used as the secondary antibody and diluted 1:1,000. The specimens were observed with a FV1200 confocal microscope equipped with a GaAsP detector (Olympus).

### Shoot Growth Analysis Following MMS Treatment

Sterilized seeds were incubated at 4°C for 24 h. The seeds were sown on 1/2 MS medium plates (1% sucrose and 0.8% agar) containing 0.05% MMS (Sigma-Aldrich). Shoot fresh weight was recorded after 14 days.

## Results

### Appearance of RAD54 Foci in Each Cell Type of Roots With DNA Double-Strand Breaks

**Figure 1:**
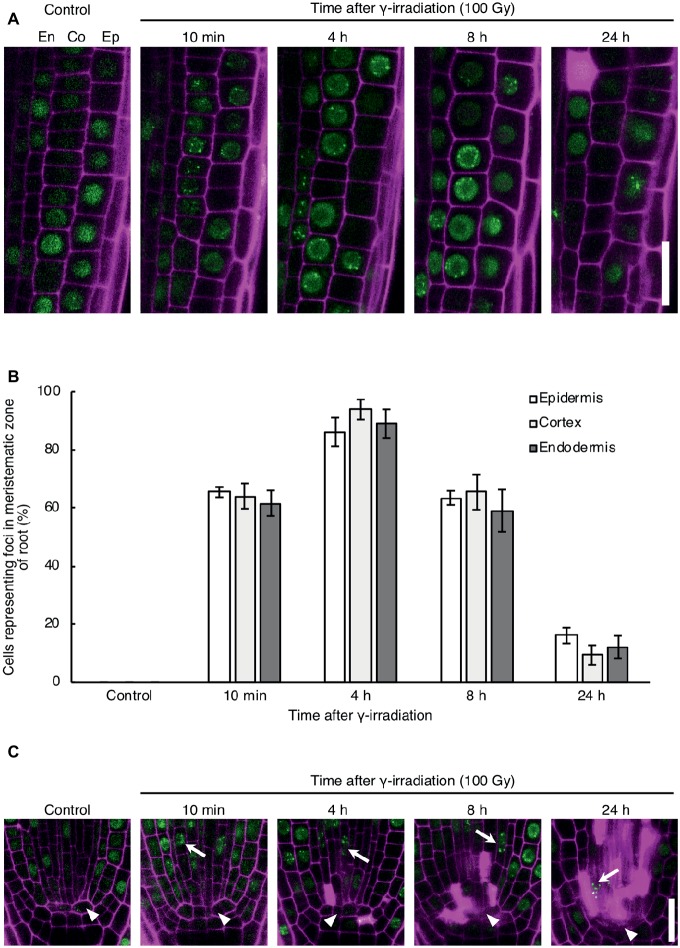
Dynamics of the formation of RAD54 foci in *Arabidopsis* root cells with DNA double-strand breaks. **(A)** Epidermal, cortical, and endodermal cells in the root meristematic zone of plants expressing RAD54-EYFP after γ-irradiation (100 Gy). Green: RAD54-EYFP. Magenta: propidium iodide. Scale bar: 20 μm. **(B)** Detection frequency of cells showing RAD54 foci in the epidermis, cortex, and endodermis in the root meristematic zone at 10 min, 4 h, 8 h, and 24 h after γ-irradiation (100 Gy). Error bars indicate the standard error. At least five roots were counted for each group. **(C)** Stem cells and quiescent center (QC) cells in the root meristematic zone of plants expressing RAD54-EYFP after γ-irradiation (100 Gy). White arrows and arrowheads indicate stem cells and QC cells, respectively. Green: RAD54-EYFP. Magenta: cell wall. Scale bar: 20 μm.

To investigate the DNA repair activity in each cell type in response to DNA damage, we observed the formation of RAD54 foci in root cells after γ-irradiation, which induces DSBs in DNA. RAD54 foci are subnuclear foci where HR repair might occur in chromatin, thus RAD54 foci can be used to monitor the activity of HR repair in living cells (Hirakawa et al., 2017). In the epidermis, cortex, and endodermis of the meristematic zone of roots, the number of cells showing RAD54 foci peaked at 4 h after 100 Gy γ-irradiation, and thereafter decreased from 8 to 24 h after γ-irradiation (Figures 1A,B). The detection frequency of cells with RAD54 foci did not differ in these cell types at each time point after γ-irradiation (Figure 1B). Thus, the HR repair activity was similar in the epidermis, cortex, and endodermis of roots with DSBs. Next, we monitored the formation of RAD54 foci in stem cells and QC cells in the meristematic zone of roots after γ-irradiation. At 10 min after γ-irradiation, stem cells showing RAD54 foci were detected in the stem cell niche, and the number of these cells increased until 8 h after γ-irradiation (Figure 1C). Stem cells with RAD54 foci were rarely observed in stem cell niches containing a greater number of dead cells at 24 h after γ-irradiation. In contrast, RAD54 foci were never detected in QC cells after γ-irradiation, which indicated that HR repair activity in QC cells differed from that in stem cells with DSBs.

### RAD54 Foci Are Detected With High Frequency During G1 or G2 Phase Cells at Long Time After Induction of DNA Double-Strand Breaks

**Figure 2:**
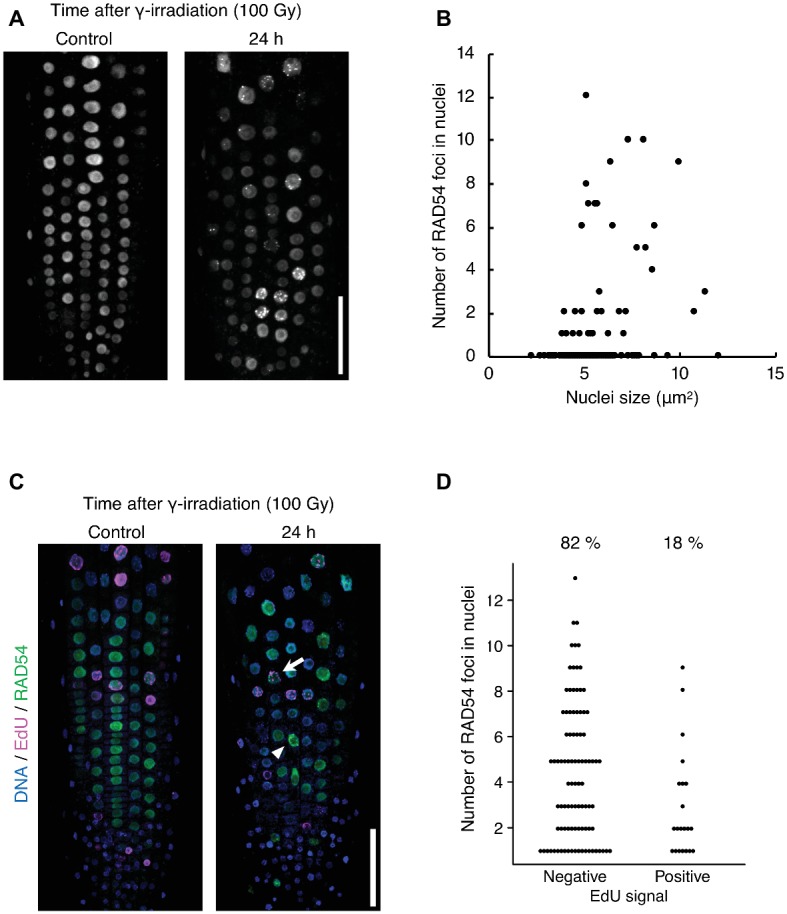
RAD54 foci are detected with high frequency during G1 or G2 phase cells at long time after γ-irradiation. **(A)** Epidermis of the root meristematic zone of plants expressing RAD54-EYFP at 24 h after γ-irradiation. Scale bar: 50 μm. **(B)** Relationship between the number of RAD54 foci in nuclei and nucleus size. The correlation coefficient is 0.28 (*p* < 0.01, *n* = 189). **(C)** Epidermis of the root meristematic zone in plants expressing RAD54-EYFP stained with DAPI and EdU at 24 h after γ-irradiation. Blue: DNA. Magenta: EdU. Green: RAD54-EYFP. White arrows and arrowheads indicate RAD54 foci positive cells labeled with EdU and RAD54 foci positive cells not labeled with EdU, respectively. Scale bar: 50 μm. **(D)** Number of RAD54 foci in cells negative and positive for EdU signals at 24 h after γ-irradiation (100 Gy). Seven roots were counted for each group. Cells lacking RAD54 foci were not counted. Upper percentages are the detection frequency of EdU-labeled cells and non-labeled cells showing RAD54 foci at 24 h after γ-irradiation. EdU negative: *n* = 93; EdU positive: *n* = 21.

The detection frequency of RAD54 foci decreased from 8 to 24 h after γ-irradiation; however, RAD54 foci were detected in each cell type except QC cells in the root at 24 h after γ-irradiation (Figures 1A–C). Thus, we characterized the RAD54 foci persisting in root cells at long time after the induction of DSBs. At 24 h after γ-irradiation, the number of RAD54 foci differed substantially among nuclei of the root epidermal cells (Figure 2A). In a previous study, we showed that most RAD54 foci were detected at high frequency in epidermal cells in the S to G2 phases of the cell cycle a short time (10 min) after γ-irradiation (Hirakawa et al., 2017). The DNA content, which increases with progression from the S phase to the G2 phase of the cell cycle, is correlated with nucleus size (Jovtchev et al., 2006). Therefore, we measured the nucleus size of cells that showed RAD54 foci to investigate the relationship between the formation of RAD54 foci and the cell cycle. At 24 h after γ-irradiation, most RAD54 foci were detected in nuclei of a wide range of sizes (4–12 μm^2^) in the epidermal cells of roots. The correlation coefficient between the number of RAD54 foci and nucleus size was low (*R*^2^ = 0.28) (Figure 2B). To further analyze the effect of cell cycle on the formation of RAD54 foci, γ-irradiated seedlings were incubated in liquid 1/2 MS medium containing EdU, which is incorporated into cells during the S phase and enables distinction between G1–G2 phase cells and S phase cells (Hayashi et al., 2013). We classified the cells showing RAD54 foci into EdU-labeled cells and non-labeled cells. In the epidermis of roots, the detection frequency of non-labeled cells was higher than that of EdU-labeled cells at 24 h after γ-irradiation (Figures 2C,D). This result might suggest that RAD54 foci formed or remained with high frequency in G1 or G2 phase cell.

### Nuclear Envelope Is Involved in Formation of RAD54 Foci With DNA Double-Strand Breaks

**Figure 3:**
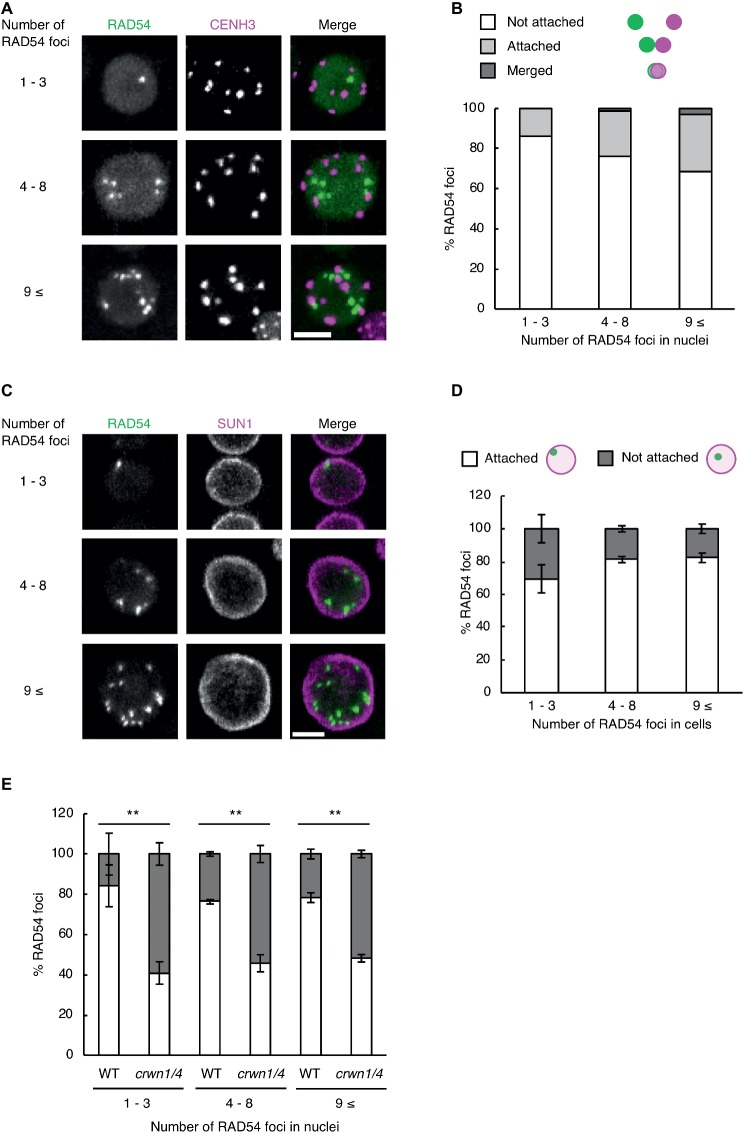
RAD54 foci attached to the nuclear periphery are detected with high frequency at long time after γ-irradiation. **(A)** Nucleus of cells in the root meristematic zone in plants expressing RAD54-EYFP and CENH3-tdTomato at 24 h after γ-irradiation. Green: RAD54-EYFP. Magenta: CENH3-tdTomato. Scale bar: 5 μm. **(B)** Detection frequency of RAD54 foci interacted with CENH3 at 24 h after γ-irradiation. The interaction pattern between RAD54 foci and CENH3 were categorized in three classes (merged with CENH3, attached to CENH3, and not attached to CENH3; *n* = 74). **(C)** Nuclei of cells in the root meristematic zone in plants expressing RAD54-EYFP and SUN1-TagRFP at 24 h after γ-irradiation. Green: RAD54-EYFP. Magenta: SUN1-TagRFP. Scale bar: 5 μm. **(D)** Detection frequency of RAD54 foci attached and not attached to the nuclear envelope (NE) at 24 h after γ-irradiation. Error bars indicate the standard error. Three roots were counted for each group. **(E)** Detection frequency of RAD54 foci attached and not attached to NE in the wild type and the *crwn1/4* double-mutant at 24 h after γ-irradiation. Error bars indicate the standard error. At least three roots were counted for each group. ^**^p < 0.01 (Fisher’s exact test). The perimeter of nuclei stained with DAPI was defined as the nuclear envelope in this experiment.

Subnuclear architecture and chromatin structure affect the efficiency of DNA repair in eukaryotes, including plants (Waterworth et al., 2011; Donà and Scheid, 2015). In cultured animal cells, the rate of DNA repair in heterochromatic regions is slower than that of euchromatic regions after γ-irradiation (Goodarzi et al., 2008). Thus, we investigated whether RAD54 foci were detected in heterochromatic regions with high frequency at long time after γ-irradiation. To visualize heterochromatic regions, we generated transgenic plants expressing RAD54-EYFP and CENH3-tdTomato, which is a centromere-specific histone H3 variant co-localized to a repetitive sequence of 180 bp present in all centromeres (Talbert et al., 2002). We classified the cells into three classes on the basis of the number of RAD54 foci (*n* = 1–3, 4–8, and 9≤; Figure 3A). In the epidermis of the meristematic zone of roots, RAD54 foci, which were merged with or attached to CENH3 signals, were rarely detected in nuclei at 24 h after γ-irradiation (Figure 3B). In cultured animal cells, the condensation of chromatin prevents the induction of DSBs from ionizing radiation (Takata et al., 2013). To check whether DSBs were induced at heterochromatic regions, we observed the formation of the histone variant γH2AX, which is the phosphorylated histone variant H2AX detected specifically at damaged sites, at chromocenters where chromatins are condensed in nuclei (Friesner et al., 2005). At 8 h after γ-irradiation, the frequency of the interaction between γH2AX foci and chromocenters was low in nuclei of the root meristematic zone (Supplementary Figure S1). These results suggested that the condensation of chromatin presented a barrier for the induction of DSBs in *Arabidopsis*. The nuclear envelope (NE) performs an important function in repairing persistent DSBs in chromatin of mammals and yeast (Gerhold et al., 2015; Amaral et al., 2017). To investigate whether the NE was involved in the formation of RAD54 foci, we generated transgenic plants expressing RAD54-EYFP and SUN1-TagRFP, which is an inner nuclear membrane (INM) protein localized to the nuclear periphery (Oda and Fukuda, 2011). The cells were classified into three classes on the basis of the number of RAD54 foci in nuclei (*n* = 1–3, 4–8, and 9≤; Figure 3C). At 24 h after γ-irradiation, more than 50% of the RAD54 foci were attached to the NE in the epidermal cells of the meristematic zone of roots (Figure 3D). To further analyze the relationship between the NE and RAD54 foci, we observed the nuclear dynamics of RAD54 in a double-mutant of the CROWDED NUCLEI (CRWN) family after γ-irradiation. The CRWN family, which are plant-specific INM proteins, function in the regulation of nuclear morphology and the arrangement of heterochromatic regions in nuclei (Sakamoto and Takagi, 2013; Wang et al., 2013). A recent study showed that a mutation in members of the CRWN family causes high sensitivity to the genotoxic agent methyl methanesulfonate (MMS) and accumulation of DNA damage following MMS treatment which suggests that the CRWN family contributes to DNA repair in response to DNA damage (Wang et al., 2019). At 24 h after γ-irradiation, the number of RAD54 foci attached to the NE in the *crwn1/4* mutant was lower than that in the wild type (Figure 3E). In addition, the *crwn1/4* mutant showed the high sensitivity to MMS relative to the WT control during shoot development (Supplementary Figure S2). These results suggested that the NE was involved in HR repair, and that CRWN1 and CRWN4 play roles in the repair of DSBs at long time after γ-irradiation in *Arabidopsis*.

## Discussion

In this study, we monitored the temporal change in appearance of RAD54 foci in *Arabidopsis* roots after γ-irradiation, to evaluate the DNA repair activity in each cell type of the root. Previous studies have reported that each cell type in roots shows a specific response to DSBs. In the epidermis and cortex, endoreduplication accompanied with an increase in cell volume is induced by zeocin, which is a DSB-inducing agent in plants (Adachi et al., 2011). In contrast, PCD was observed specifically in the stem cells of root tips in response to zeocin treatment (Fulcher and Sablowski, 2009). The present microscopic analysis showed that the detection frequency of cells with RAD54 foci was not significantly different in the epidermis and cortex at each time point of observation after γ-irradiation (Figures 1A,B). In addition, the pattern of stem cells with RAD54 foci detected after γ-irradiation was similar to that in the epidermis and cortex (Figure 1C). These results suggested that RAD54-dependent HR repair occurred at the same frequency in the epidermis, cortex, and stem cells, whereas these cells showed different responses to DSBs. In stem cell niches, cells showing RAD54 foci and cells undergoing PCD were detected after γ-irradiation (Figure 1C). Given that the PCD pathway is closely associated with the signaling pathways in response to DNA damage, PCD might affect the formation of RAD54 foci (Nowsheen and Yang, 2012). Although the signaling pathways activated following DNA damage in plants have been studied in detail, the mechanism controlling PCD in response to DNA damage is still unclear (Yoshiyama et al., 2013b). A number of nucleases and proteases, such as *BIFUNCTIONAL NUCLEASE 1* and *CYSTEINE ENDOPEPTIDASE 1*, could be used to visualize the PCD process in plants (Farage-Barhom et al., 2008; Zhang et al., 2014). Thus, it might be possible to reveal the relationship between the formation of RAD54 foci and PCD by dual fluorescence imaging of RAD54 and these markers of PCD. Interestingly, RAD54 foci were not detected in QC cells at each time point after γ-irradiation (Figure 1C). This result is consistent with the observation that progression of the cell cycle in QC cells is arrested at the G1 phase when HR repair activity is low owing to the absence of sister chromatids (Forzani et al., 2014). There are findings about the mechanisms to maintain genome stability in QC cells of animals. In hematopoietic stem cells of mice, non-homologous end-joining mediated repair but not HR repair is preferentially used for repair of DNA damage during the quiescence phase (Mohrin et al., 2010). The detection frequency of γH2AX foci induced by heat stress in quiescent human endometrial mesenchymal cells (MSCs) is considerably lower than that in proliferating MSCs (Alekseenko et al., 2018). Thus, it is suggested that the mechanism of DNA repair in QC cells also differs substantially from that in differentiated cells and stem cells in plants.

We found that RAD54 foci were detected with high frequency during G1 or G2 phase cells in roots at 24 h after γ-irradiation (Figure 2D). This result indicates the possibility that RAD54 formed or remained in these cells at long time after the induction of DSBs. To address this question, it might be effective to monitor the appearance and disappearance of RAD54 foci in nuclei after γ-irradiation by time-lapse imaging of RAD54. Additionally, the visualization of G1 and G2 phase cells could definitely reveal the close relationship between these phases and RAD54 foci at long time after γ-irradiation. We also observed that cells showing RAD54 foci persisted in roots and that RAD54 foci attached to the NE were detected with high frequency in these cells at 24 h after γ-irradiation (Figures 3C,D). In *Drosophila*, DSBs in heterochromatic regions move to the nuclear periphery to complete HR repair, and the defect of anchoring DSBs at the nuclear periphery reduces tolerance to γ-irradiation and induces ectopic recombination, which might occur between repetitive sequences in heterochromatic regions (Chiolo et al., 2011; Ryu et al., 2015). Persistent DSBs induced by the budding yeast HO-endonuclease system are relocalized to the nuclear periphery, where the DSBs directly bind to the Nup84 nuclear pore complex (Nup84, Nup120, and Nup133) and the INM protein Mps3 (Nagai et al., 2008; Kalocsay et al., 2009). In addition, the budding yeast mutants of Nup120 and Mps3 show high sensitivity to MMS and unequal exchange of sister chromatids (Horigome et al., 2014). The present *Arabidopsis* mutant analyses showed that the plant-specific INM proteins CRWN1 and CRWN4 are required for attachment of RAD54 foci to the nuclear periphery at a long time after γ-irradiation (Figure 3E). Thus, we suggest that the NE contributes to the progression of HR repair in eukaryotes, and that CRWN1 and CRWN4 are involved in NE-mediated HR repair and maintenance of genome stability in response to DSBs in plants.

## Data Availability

The datasets for this manuscript are not publicly available because our manuscript does not include sequence data sets, for example, RNA-seq and ChIP-seq. Requests to access the datasets should be directed to sachi@rs.tus.ac.jp.

## Author Contributions

TH and SM designed this research and wrote the manuscript. TH performed all experiments.

### Conflict of Interest Statement

The authors declare that the research was conducted in the absence of any commercial or financial relationships that could be construed as a potential conflict of interest.

The handling editor declared a past co-authorship with one of the authors SM.

## Supplementary Material

SUPPLEMENTARY FIGURE S1 γH2AX foci are not detected at chromocenters after γ-irradiation. **(A)** Nucleus showing γH2AX foci in the root meristematic zone at 8 h after γ-irradiation. Green: γH2AX. Magenta: DNA. Scale bar: 5 μm. **(B)** Detection frequency of γH2AX foci interacted with chromocenters at 8 h after γ-irradiation. The interaction pattern between γH2AX foci and chromocenters were categorized in three classes (merged with chromocenters, attached to chromocenters, and not attached to chromocenters; *n* = 49).

SUPPLEMENTARY FIGURE S2 *crwn1/4* mutants show high sensitivity to MMS during shoot development. **(A)** Shoot development of wild type and *crwn1/4* plants treated with and without 0.05% MMS. Scale bar: 0.5 cm. **(B)** Shoot fresh weight of WT and *crwn1/4* plants treated with and without 0.05% MMS (*n* = 20). *p* < 0.01 (Student’s *t*-test).
