# Supplementary figures and images for: Characterization of DNA Repair Foci in Root Cells of Arabidopsis in Response to DNA Damage
Source: Front Plant Sci. 2019 Jul 30;10:990. doi: 10.3389/fpls.2019.00990 (PMC6682680; doi:10.3389/fpls.2019.00990)

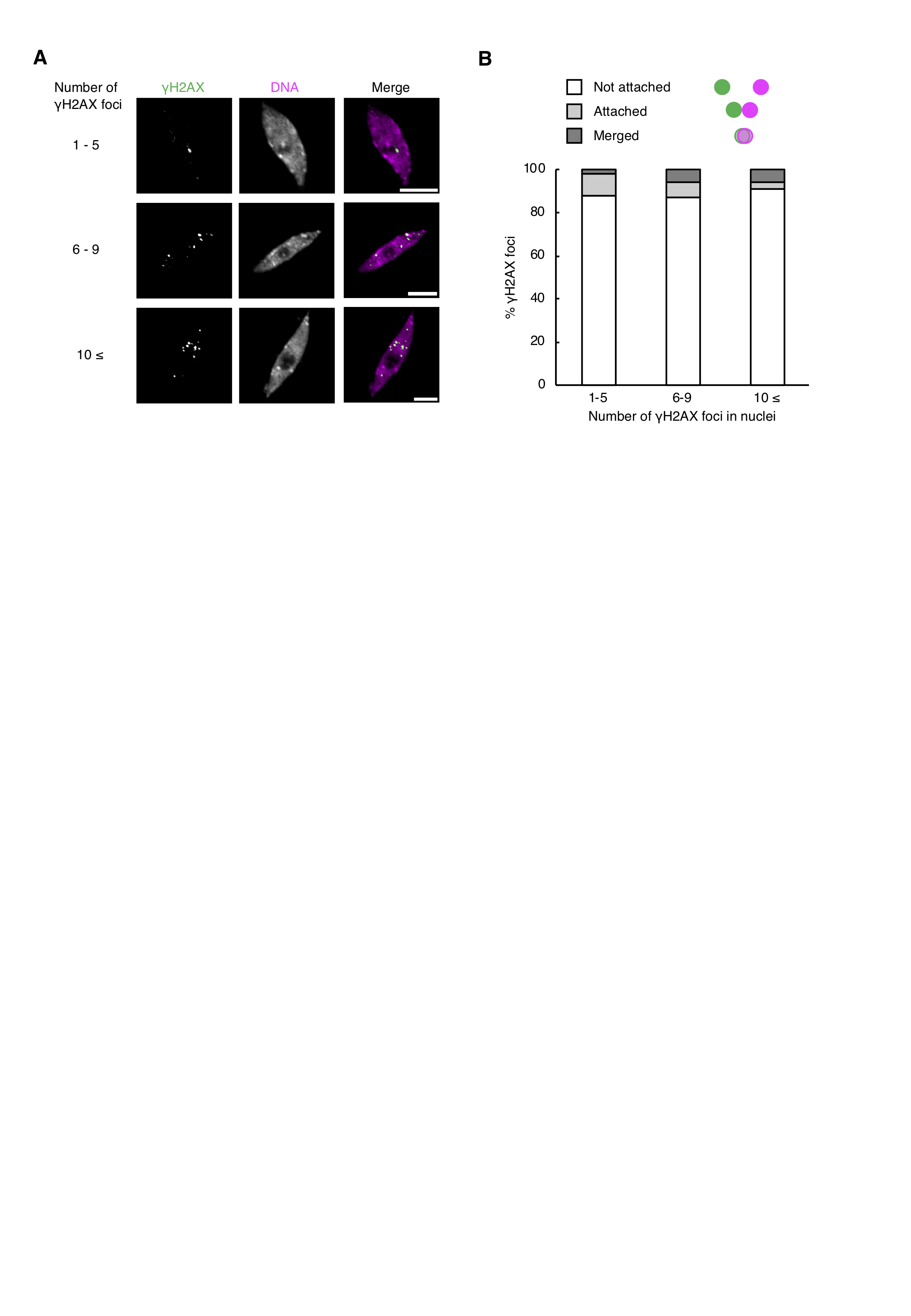

Supplement: SUPPLEMENTARY FIGURE S1 — γH2AX foci are not detected at chromocenters after γ-irradiation. (A) Nucleus showing γH2AX foci in the root meristematic zone at 8 h after γ-irradiation. Green: γH2AX. Magenta: DNA. Scale bar: 5 μm. (B) Detection frequency of γH2AX foci interacted with chromocenters at 8 h after γ-irradiation. The interaction pattern between γH2AX foci and chromocenters were categorized in three classes (merged with chromocenters, attached to chromocenters, and not attached to chromocenters; n = 49). [file Image_1.TIFF]

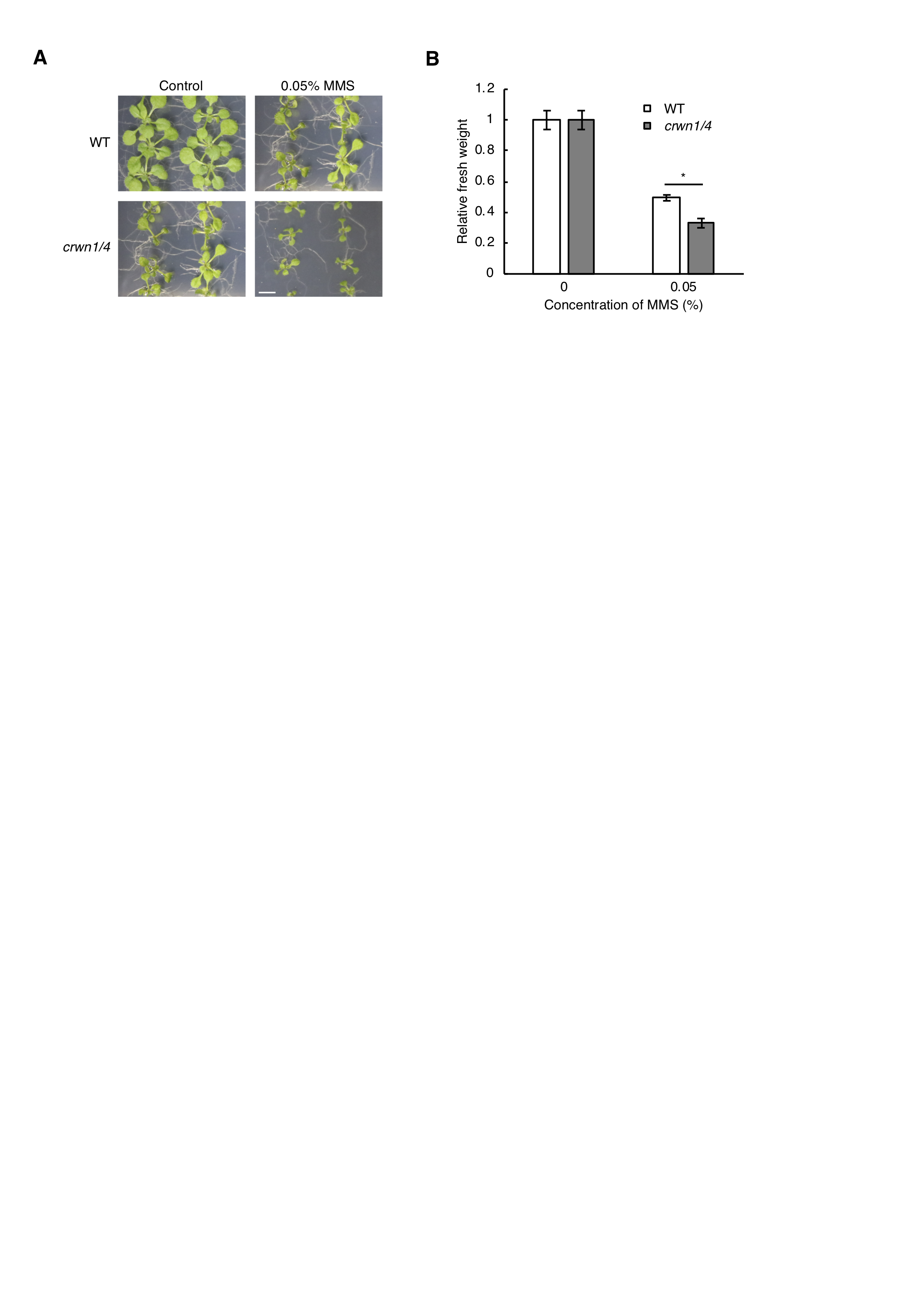

Supplement: SUPPLEMENTARY FIGURE S2 — crwn1/4 mutants show high sensitivity to MMS during shoot development. (A) Shoot development of wild type and crwn1/4 plants treated with and without 0.05% MMS. Scale bar: 0.5 cm. (B) Shoot fresh weight of WT and crwn1/4 plants treated with and without 0.05% MMS (n = 20). p < 0.01 (Student’s t-test). [file Image_2.TIFF]
